# Special Anatomy and Histology

**Published:** 1851-11

**Authors:** 


Art. VII.—*Special Anatomy and Histology.* By Wm. E. Horner, M.D., Professor of Anatomy in the University of Pennsylvania, etc., etc. Eighth edition. Illustrated with anatomical plates. Philadelphia: Blanchard & Lea.

This work has long held a distinguished rank among the elementary works on the science of Anatomy. We know of no work, indeed, superior to this for the use of students. Its descriptive powers are remarkably well exhibited, and its details are given clearly and forcibly.

The edition before us, the eighth, has received some additions in order to bring the work up to the present advanced state of anatomical science, and there is a considerable increase, in this edition, of the illustrations. A great many of these illustrations are admirably executed. But it is unnecessary for us to dwell on the merits of a work that has long enjoyed the universal approbation of the profession, and which is not likely soon to be displaced from its high and well merited position. We commend to the study of young men about entering the medical profession, the eighth edition of Horner's Special Anatomy; if well studied it will be found in the highest degree serviceable. The publishers have performed their duty faithfully in the mechanical department. B.

